# P14 Surgical source control and colistin therapy for the management of osteomyelitis due to *Pseudomonas aeruginosa* with difficult-to-treat resistance

**DOI:** 10.1093/jacamr/dlad066.018

**Published:** 2023-06-14

**Authors:** V Nageshwaran, J Ferguson, A Ramsden, L Barrett, M Scarborough, T Wangrangsimakul, M Morgan, S Oakley, B C Young, N Stoesser

**Affiliations:** Oxford University Hospitals NHS Foundation Trust, Oxford, UK; Oxford University Hospitals NHS Foundation Trust, Oxford, UK; Oxford University Hospitals NHS Foundation Trust, Oxford, UK; Oxford University Hospitals NHS Foundation Trust, Oxford, UK; Oxford University Hospitals NHS Foundation Trust, Oxford, UK; Oxford University Hospitals NHS Foundation Trust, Oxford, UK; Oxford University Hospitals NHS Foundation Trust, Oxford, UK; Oxford University Hospitals NHS Foundation Trust, Oxford, UK; Oxford University Hospitals NHS Foundation Trust, Oxford, UK; Nuffield Department of Medicine, University of Oxford, Oxford, UK; NIHR-Oxford Biomedical Research Centre, Oxford, UK; Oxford University Hospitals NHS Foundation Trust, Oxford, UK; Nuffield Department of Medicine, University of Oxford, Oxford, UK; NIHR-Oxford Biomedical Research Centre, Oxford, UK

## Abstract

**Patient:**

A 45-year-old man from Lagos, Nigeria, now living in the UK, presented to his local hospital with purulent discharge from his knee. During a road traffic accident in 2019, he had sustained open fractures of his femur and tibia. These required surgical fixation and he underwent four surgeries over a 10-month period in Nigeria. He was reportedly on antibiotics throughout this period. In early 2022, he noticed discharge from the knee and was referred to the Bone Infection Unit in Oxford. On examination, left leg shortening and a sinus on the anterior proximal tibia were noted. Knee flexion was reduced to 30 degrees. He was otherwise systemically well. PET-CT revealed two areas of avidity, supporting the clinical diagnosis of fracture-related infection adjacent to a retained tibial plate.

**Treatment:**

The patient underwent removal of the tibial plate, excision of infected bone, insertion of local antibiotic carrier (Cerament G with vancomycin added) and a medial gastrocnemius flap with split skin grafting. Five out of five intra-operative samples were culture positive for *Pseudomonas aeruginosa*, with NDM metallo-β-lactamase detected. This was resistant to ceftazidime, meropenem, piperacillin/tazobactam, gentamicin, tobramycin, amikacin, ciprofloxacin, ceftazidime/avibactam, imipenem/cilastatin/relebactam, ceftolozane/tazobactam and cefiderocol. The organism had an aztreonam MIC of 16 mg/mL and a colistin MIC of 2 mg/mL. The patient also had MRSA isolated from all intra-operative samples. Following multidisciplinary team discussion, he was started on aztreonam 2 g QDS and colistin loading with 9 million IU followed by 4.5 million IU BD for the *P. aeruginosa* and doxycycline 100 mg BD for the MRSA. After 8 days of therapy, he developed a symmetrical body rash, which was felt to be a delayed hypersensitivity reaction to aztreonam and resolved when aztreonam was stopped. He was subsequently treated with colistin monotherapy for 6 weeks via OPAT. Whilst on this treatment, his renal function was monitored regularly with appropriate dose reductions because of renal impairment. Follow-up at 9 months demonstrates good recovery with no pain or symptom recurrence.

**Conclusions:**

This case illustrates the challenges of managing fracture-related infections with extensively drug-resistant organisms and the importance of multi-disciplinary input. Drug-resistant infections requiring prolonged courses of antibiotics can be successfully managed using an OPAT service. Finally, this case emphasizes the importance of understanding global patterns of antimicrobial resistance and stewardship to identify cases that are at risk of being infected with drug-resistant bacteria especially where there may be long intervals between establishment of the infection and presentation to healthcare services.

